# Exploring the Feasibility of a Bracketing Approach Utilizing Modeling for Development of Long-Acting Injectables for Regulatory Approval—A Case Study Using Levonorgestrel

**DOI:** 10.3390/ph17121640

**Published:** 2024-12-06

**Authors:** Susan Cole, Henry Pertinez, Andrew S. Butler, Essam Kerwash, Swati Bhat, Eman El-Khateeb, Andrew Owen

**Affiliations:** 1 Medicines and Healthcare Products Regulatory Agency, Canary Wharf, London E14 4PU, UK; 2 Centre of Excellence for Long-Acting Therapeutics University of Liverpool, Liverpool L69 3BX, UK; 3 Clinical Pharmacy Department, Faculty of Pharmacy, Tanta University, Tanta 31527, Egypt

**Keywords:** contraceptives, long-acting, exposure response, PBPK

## Abstract

Background: The development of long-acting products of a characterized drug substance is of great interest. It is possible to support the development of these products with available clinical data by matching the exposure to a predefined bracket of a minimal concentration for efficacy and a maximal concentration for safety. This bracketing approach would cut down on the time and cost of new long-acting contraceptive products progressing to market. The current study describes the assessment of the data available to support a bracketing approach to conclude comparable levels of efficacy and safety for a postulated novel long-acting reversible contraceptive (LARC) product of levonorgestrel. Methods: Literature evidence of levonorgestrel efficacy, as quantified by the Pearl Index, was utilized and modeled by incorporating three LARC products for the estimation of a minimal concentration required for efficacy. Further literature was reviewed to quantify the maximal concentration required to ensure product safety. Additionally, a review of the regulatory precedence for the approach was conducted using European and UK databases. Results: There was a reasonable definition of the minimal concentrations for efficacy where the target concentrations of levonorgestrel were in the range of 200–400 pg/mL. Maximum concentrations for safety were less well defined. Although regulatory guidance supports the bracketing approach, there is little precedence for licensing new products based on pharmacokinetic data only, despite much reduced clinical and non-clinical packages being evidenced. Conclusions: Understanding of the exposure response is not currently considered sufficient to support a bracketing approach for a new levonorgestrel product. If additional safety data are established, current regulations may allow for a reduced application package. Additional work is needed to support the approach, and this could utilize the wealth of information in real-world datasets combined with systems models.

## 1. Introduction

For well-known and established drug substances, the drug development program of an alternative product formulation may rely on the known relationships between exposure and efficacy as well as exposure and safety [1,2]. Similarly, prior information from non-clinical studies may be used to reduce the need for animal studies by considering the known exposure–response (ER) relationship for toxicity. Modified-release products of an already marketed immediate release or an alternative modified release require comparative clinical efficacy and safety data in addition to pharmacokinetic data [3]. However, if there is a well-defined relationship between plasma concentrations of the active substance and the clinical response, clinical efficacy and safety trials may be considered unnecessary and can be substituted by pharmacokinetic studies.

The European Medicines Agency (EMA) has produced guidance on the development of modified-release formulations, which are applicable to long-acting injectables [1]. In the modified-release guidance, the following are recommended:

*“As a principle, comparative clinical efficacy and safety data are needed in addition to PK data for modified-release products developed after the immediate-release formulation unless adequately justified. As the efficacy and safety of the immediate-release product are known, the major issue would be to demonstrate that the new modified-release formulation is as safe and effective as the existing formulation. However, in exceptional cases, if the assessment of the concentration–effect relationship indicates that there is a well-defined relationship between plasma concentration(s) of the active substance/active metabolite(s) and clinical response, clinical trials may be considered unnecessary. In this case, the same or a better level of efficacy and safety has to be concluded from PK/PD studies.”*

Once a therapeutic window has been defined with regard to safety and efficacy, pharmacokinetic modeling could help to keep the exposure inside this window.

The development of long-acting products is of growing interest across many therapeutic areas due to the increased convenience, protection of healthcare privacy, and overcoming challenges with adherence to medication. Currently, long-acting medicines for cancer [4], malaria, tuberculosis (TB), hepatitis C, and HIV are being investigated [5]. Machine learning models were utilized to accelerate the design and development of polymeric long-acting injectables [6]. The development of long-acting injectables has been of interest for over 40 years, and specific guidance for antipsychotic drugs is available [7]. For drugs such as contraceptives, where even short interruptions in drug intake can significantly affect efficacy, long-acting formulations may be particularly advantageous [8].

If an exposure-based approach is used in the development of alternative hormonal contraceptive products of a well-characterized active substance (e.g., one that has been in marketed products for several decades), efficacy may be assumed when a new product maintains exposures at or above the concentrations known to ensure the efficacy of approved products. Safety and tolerability may be assumed when both the Cmax and AUC exposures for the new product remain at, or below, the exposures for approved products. These concentrations, if known, can be considered to define brackets within which exposure should be maintained [9].

The public health consequence of unintended pregnancy is greatest in countries where maternal mortality and morbidity are high. Unintended pregnancy is often attributed to sexual activity without the use of contraception. However, inconsistent use remains a significant cause of unintended pregnancies [10]. Short-acting contraceptives depend on user adherence and, therefore, have higher failure rates in typical use compared to perfect use (e.g., oral contraceptives have a failure rate of 9% for typical use vs. 0.3% for perfect use [11]). By contrast, the effectiveness of long-acting reversible contraceptive (LARC) methods does not depend on daily adherence. LARCs can be defined as contraceptive methods that require administration less than once per cycle or month [12]. The use of LARCs is low in Great Britain, at around 12% of women aged 16–49 in 2008–2009, compared with 25% for the oral contraceptive pill and 25% for male condoms [13]. Included in the category of LARCs are copper intrauterine devices, progestogen-only intrauterine systems, progestogen-only injectable contraceptives, and progestogen-only subdermal implants.

Alternative contraceptive products with a variety of durations of action are desired to improve user adherence and better health outcomes. Such products often use drug substances (or active pharmaceutical ingredients, API) of already marketed oral dosage forms with alternative delivery systems as they are supported by a substantial amount of safety and efficacy data. There is guidance on the clinical investigation of steroid contraceptives in women [14].

Levonorgestrel (LNG) is a progestogen used in both routine and emergency contraception and as hormone therapy for symptoms of menopause [15]. It is available in several formulations, including oral, intra-uterine, transdermal gels, or subcutaneous implants. Characterization of the safety and efficacy of contraceptives is dependent upon reporting/recording of adverse events and pregnancies. Pregnancy rates are typically calculated using the ‘Pearl Index’ (PI) [16], a statistical method developed in the 1930s to compare contraceptive failures across different methods of contraception. The Pearl Index determines the number of pregnancies per 100 women years. Typically, it is calculated using trial data, where the use of the contraceptive method is closely controlled. LNG is perhaps the best characterized of the hormonal contraceptives in terms of its pharmacokinetics. Thus, it serves as a useful case study in evaluating the plausibility of using a bracketing approach to streamline the development of new LARCs

This paper describes the assessment of the data available to support a bracketing approach to conclude the same or better levels of efficacy and safety for a postulated novel long-acting contraceptive product of levonorgestrel to support the rapid development of these products and facilitate regulatory approvals. This is based on the literature evidence of the efficacy and safety utilizing modeling of the data and includes a review of European and UK regulatory databases for evidence of precedence of this approach.

## 2. Results

### 2.1. Defining the LNG Exposure Bracket

#### 2.1.1. Defining the Efficacy Concentrations

**Figure 1:**
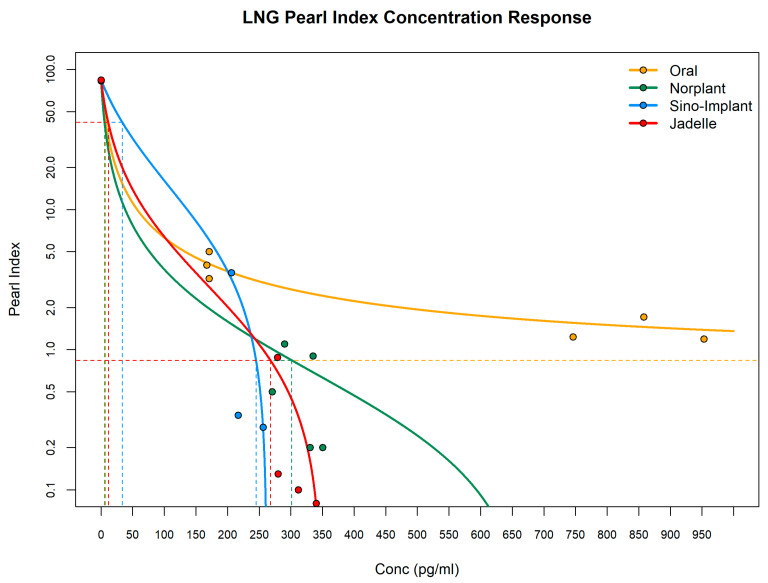
Imax model fittings to LNG Pearl Index concentration–response relationships. Reference lines were drawn for concentrations giving 50% and 99% of the baseline Pearl Index.

The data retrieved (with digitization of figures where necessary) are summarized in Table 1 for Norplant [17,18], Sino Implant [19], and Jadelle [20,21], with plots of the data and Imax model fittings in Figure 1. Figure 1 also includes reference data and an Imax model fitting for an oral LNG PI vs. concentration–response curve derived from Lingineni et al. (2021) [22], which provides the important anchoring baseline PI of 84% at a zero LNG drug concentration (i.e., PI in the absence of LNG contraception, reflecting baseline fertility of the population combined with the random potential use of other contraceptive methods). This baseline PI value is commonly used across all the concentration–response curves herein. In Figure 1, the non-convex appearances of some curves (e.g., Sino-Implant and the Jadelle curves) are due to the logarithmic scale on the *y*-axis of the plot combined with the specific values of the Imax and IC50 parameters.

It should be noted that while the PI can only be calculated over a time window (usually of at least several months), PK concentrations (that are being considered to drive the matching timepoint PI effect) are generally reported for a specific single timepoint. For the oral profile, reported concentrations assume steady-state exposure (under repeated dosing) over the window of PI determination at different dose levels. For the implant profiles, the very slow release of LNG from the implant (leading to flip-flop pharmacokinetics [23] and a long, slow apparent terminal half-life in the PK profile following implantation) somewhat mitigates this issue, assuming the reported concentration driving the reported PI drops only a small amount (i.e., is approximately constant) over the window of PI determination.

The target concentrations for efficacy of levonorgestrel appear to be in the range of 200–400 pg/mL, and this range corresponds with the estimated brackets for efficacy. These concentrations are consistent with exposures (inclusive of population variability) previously seen for the LNG implant dosing route [24,25]. There is an apparent divergence between implant and oral data, with reduced efficacy of oral forms implied at higher concentrations.

#### 2.1.2. Defining the Safety Concentrations

The literature reviews using (“exposure-response” AND “levonorgestrel”) as a search term show very limited results, with only one study identified [26] which investigates the effects of inducers on the PK of contraceptives and hence the focus on efficacy rather than safety. More literature was identified using the term “exposure-response for the safety of hormonal contraceptives” based on specific adverse events of interest, such as weight gain, bleeding, or change in bone mineral density. There are a number of comprehensive reviews of the efficacy and safety of contraceptives [27,28].

In general, over many years of use, no serious safety signals have emerged for LNG implants, although some side effects have been noted. Since healthy young women take these medications, it is important that the drugs are safe. Weight gain, irregular bleeding, and bone density loss have been identified as possible safety concerns. Analysis of safety data, however, is complicated by region-specific aspects of data collection and reporting, poor adherence, differences in pharmacokinetic profiles from the products with different routes of administration (e.g., daily oral versus long-acting implants), and interactions with co-administered products [29,30]. The use of integrated quantitative modeling methods has been proposed to help bridge some of these gaps [8,9].

There is contradictory information in the literature on the effect of hormonal contraceptives on weight gain, and this is complicated again by the different products available. Generally, no statistically significant weight gain is seen for levonorgestrel implants [31]. While some differences between substances and products have been documented, there is no evidence of an exposure-response understanding of levonorgestrel [32].

Breakthrough bleeding (BTB) is the most common side effect while on hormonal contraceptives and a primary reason for discontinuation. The challenges associated with its assessment include inconsistent terminology across clinical trials, difficulty reporting/capturing bleeding events, and a lack of standardized classification, statistical methods, and dose-response data. A model-based meta-analysis to capture the BTB dose response illustrated that BTB is affected by progestin type and the dose of ethinyl estradiol, which provides protective effects against BTB [33]. Efforts are ongoing to correlate this to the concentration of individual products. Real-world data (RWD) indicated that BTB events were more frequent in women taking depot medroxyprogesterone acetate (DMPA). This could be due to a higher instance of reporting since women can report side effects when they are presenting for their next injection. Women on other contraceptive methods, however, do not have that healthcare visit as an opportunity to report side effects [34]. Currently, there is no good understanding of the exposure and BTB for any progestins.

Effects on bone mineral density (BMD) are a concern, particularly in adolescents, where there is a rapid period of bone mass accrual. This is important as any drug treatment that is associated with potential bone loss can cause a blunting effect on bone mass accrual in adolescence and subsequently alter the trajectory toward reaching peak bone mass. The use of progestin-only contraceptives in adolescents has shown a decrease in BMD and an inconclusive but potential concern of blunting the peak bone mass. DMPA specifically has evidence of BMD decreasing with use in adolescents, but BMD has been shown to be restored after discontinuation of DMPA [35]. The associated fracture risk is inconclusive. However, long-term DMPA use (>30 months) tends to be associated with increased fracture risk [30]. There is strong evidence that DMPA changes the BMD, and there is a black box warning on the product label [36]. The effect of other progestins on BMD is unclear, and it is suggested that this may be due to a lack of rigorous study [37]. It is unclear how much correlation can be made from the data about bone mineral density and fracture risk prediction as there is no solid evidence regarding the two. Methodologies might not be comparable across studies since some studies used bone density (DEXA) scanning whilst others used bone density measurement of different bones.

In general, the concentration–effect relationships for safety or adverse events do not appear to be well established in the literature and are technically very challenging to define, particularly considering the variability of the compound. The current data does not allow the setting of a maximal concentration or upper bracket for safety.

### 2.2. Regulatory Precedence for the Approach

Searches of the EMA and MHRA websites based on the search ‘long-acting injectable’ or on specific contraceptive products were the most productive. These identified Xeplion/Trevicta/Invega, Okedi, Zyphadera, Abilify Maintena, Rekambys, Prostrap, Buvidal, Exparel, and Bydureon [38,39,40,41,42,43,44,45,46,47,48,49,50,51,52,53]. These examples were generally submitted as a hybrid application (EMA 10(3)/MHRA 52) to an application by reference to data for the same product by a different dose route or a stand-alone application (EMA 8(3) mixed/MHRA 50), also including the literature data generated utilizing a different dose route.

**Figure 2:**
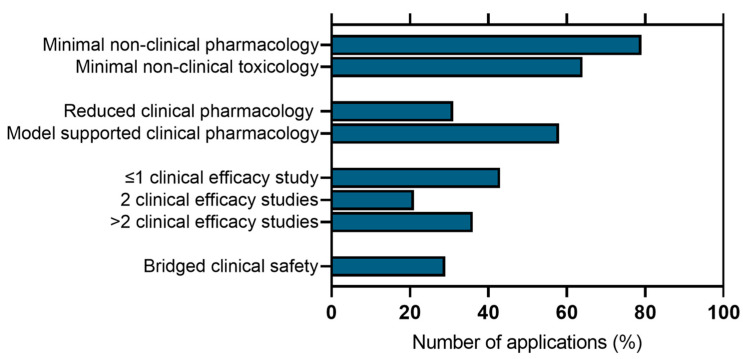
Summary of the application packages identified.

Of the long-acting injectable data collected, a number of these were antipsychotic medicines (e.g., paliperidone, risperidone, olanzapine, and aripirazole). In addition, data was collected for the antiviral, rilpivirine, used with cabotegravir in the long-acting combination for HIV, leuprorelin (oncology), buprenorphine (opioid dependence), bupivacaine (anesthesia), and exenatide (type 2 diabetes). Data was also collected for Zutectra (hepatitis) and two contraceptives of interest, DMPA injections and levonorgestrel IUDs. A summary of the data is shown in Figure 2, and these data are detailed in Table 2.

In most cases, limited or no non-clinical pharmacology studies were performed for the new product. Very few examples included new pharmacology or PK studies, and instead, evaluation relied on data generated from a different dose route, especially studies under good laboratory practice (GLP). Supporting ADME studies (e.g., mass balance studies) also tended to rely on the available data from different dosing routes, but studies were performed to characterize the pharmacokinetics by the intended dose route.

Limited toxicology studies in non-clinical species were also performed, again relying on data from the alternate dosing route. Specific studies usually involved local tolerance studies. In some cases, more extensive toxicity packages were provided (e.g., at 6 months), and these usually supported applications where there was a more extensive difference in profile between the new product and the reference. For example, Xeplion, where the reference is a short-acting oral product, and the new product is an injectable with a considerably increased duration of action.

Clinical Pharmacology studies were seen to be important in these applications and are generally used to bridge exposure between products by comparison of PK profiles. Population (PKPD) models are used in a number of cases to support the proposed posology (paliperidone, risperidone, aripiprazole, buprenorphine, leuprorelin, and levonorgestrel). In vitro in vivo correlation (IVIVC) models are also used in some examples to support differences in the release rate (paliperidone and leuprorelin) or to extend the duration of action (Mirena).

Some form of clinical efficacy study is provided in every application, with the exception of those in which the change is very minor (e.g., injection device for DMPA). In most cases, two phase III efficacy studies are performed, but in some cases, one phase III study appears to be acceptable (buprenorphine, exenatide, leuprolide, and zutrecta). When only one phase III study is performed, this is typically supported by clinical pharmacology modeling (e.g., buprenorphine). A comparator is not always included (Zutectra, Sayana, and Mirena).

Safety data to support the application is usually generated in the clinical studies used to establish efficacy, but this is often in a limited number of subjects, and bridges have been made to data from other products.

As an example, in psychiatry, the EMA’s approval of the original oral tablet aripiprazole (Abilify) [38] for the treatment of patients with schizophrenia was based on three positive (vs. placebo) pivotal phase III clinical trials involving more than 1200 subjects in total. The subsequent EMA approval of a long-acting injectable lyophilized product of aripiprazole (Abilify Maintena) was based on these prior efficacy results established from trials with the oral formulation and the efficacy of the new product in maintaining symptomatic control in a phase III trial with 196 subjects completing the depot 400/300 mg arm, 178 subjects completing the 10–30 mg oral arm, and 61 subjects completing the 50/25 mg depot arm of the study (total number of subjects randomized was *n* = 662). Specifically for the contraceptives, the initial development of the DMPA intramuscular depot was based on the oral product and had a duration of effect of 13 weeks. The application was supported by two phase III efficacy and safety studies, which were open-label, non-comparative of 1 year duration. Additional collection of safety data was ongoing at the time of authorization [39]. For Mirena, to extend the duration of action from 5 up to 8 years, the applicant conducted a study to assess the contraceptive efficacy during extended use in 362 women in a US-Mirena Extension Trial. This was also supported by simulations using an established IVIVC and the population PK analysis of the levonorgestrel-releasing intrauterine system over 8 years and in comparison with other levonorgestrel-containing products. Safety data was also generated in the efficacy study [51,52,53].

## 3. Discussion

The regulatory review shows several examples where long-acting injectables or implants have been developed for known active substances by bridging the data from other clinical developments of the same substance. Consequently, savings are possible in the non-clinical and clinical data required. In general, the non-clinical data to support these applications relies heavily on the known information and is often restricted to only local tolerance studies. Pharmacokinetic studies are important to allow the bridging of data across different products, and population models are extensively used to support the bridge and the proposed posology. Clinical efficacy data consisting of two-phase III studies is used to support the application; however, in some cases, an abbreviated package can be used (e.g., modeling to support the extension of the duration of use of Mirena). Safety data can also be bridged from the previously established data.

There are no examples to date of licensing a new long-acting product based solely on PK and an established E-R for efficacy and safety or of the utilization of the bracketing approach, which would use the knowledge of the efficacy and safety concentrations to maintain concentrations in a pre-defined bracket. This approach, based on a well-characterized exposure response, has, however, been used to support the development of alternative release products (e.g., tofacitinib modified release clinical package relied solely on PK). This demonstrated a similar overall profile, but a 29% lower Ctrough was observed when the dose was changed from the 5 mg immediate-release formulation given twice daily to the 11 mg extended-release formulation given once daily [40].

The application of exposure-response modeling showed that Cave was the most relevant parameter for efficacy, and the 29% lower Ctrough was not likely to be clinically important to predict efficacy for tofacitinib. Therefore, no additional efficacy studies were required.

In consideration of such an approach for levonorgestrel, the target concentrations for efficacy of levonorgestrel appear to be in the range of 200–400 pg/mL, and this is consistent with the prolonged efficacy seen for implants observed in the literature [20]. The 200 pg/mL, therefore, appears to be the lower bound that should not be crossed while developing a new LNG LARC. This value is below IC_99_ across all products, and it is suggested that the 5th percentile of a PK trial should be maintained at least above this value.

An important limitation of this analysis is that the concentration-response profiles are somewhat sparse and show a degree of variability that makes the fitting of the I_max_ curves challenging and somewhat imprecise. Noise and variability in the data, for example, lead to Imax estimates slightly greater than 100%, and there is a lack of characterization of the concentration-effect relationship in the lower efficacy (higher PI) region of the curves. Despite any limitations with the model fittings and the precision of parameter estimates, it is apparent, even from the observed data alone, that there is a leftward shift in the concentration-response data for implants compared to the relationship for oral LNG. As PI efficacy is assessed/modeled vs. concentration rather than dose, it is possible to assume that the concentration reported for driving implant efficacy might have a different context compared to concentrations reported for driving oral PK/PD. The reason is that, for implant PK, no assumptions of steady-state exposure and accurate adherence to the dosing regimen are required, and there is no peak-to-trough variability associated with each dose (there is only a single implant dose followed by a prolonged slow, flip-flop PK release exposure profile). It might, therefore, be the case for the oral LNG that higher apparent doses and exposures appear to be required to achieve similar efficacy because this is needed to mitigate the lower exposures due to incomplete adherence [30] and lower trough concentrations. Meanwhile, both oral and subdermal implant LNG dose routes will have higher apparent systemic exposures required for efficacy than, for example, an intrauterine contraceptive implant able to achieve targeted local exposure more precisely at the site(s) of efficacy [25].

Another limitation is that the exposure-response (ER) for safety is not as well defined for any of the events identified to be of interest (weight gain, BTB, and BMD). It appears technically very challenging to define the ER for any of the AEs due to the extremely low incidence of the effects and the absence of correlated PK data. It may, in fact, be prohibitive to prospectively design a trial to intentionally push the dose higher to see such effects and to collect adequate data for effects that take a long time to develop.

Additional information is required before a maximal ‘safe’ concentration can be agreed upon, and additional events such as venous thromboembolism will also need to be considered [55]. This additional knowledge should include a better understanding of the mechanisms underlying safety concerns. For example, changes in BMD could potentially be informed and predicted by incorporating in vitro binding affinity data into system pharmacology models.

There is also a wealth of “real world data” (RWD) potentially able to inform both safety and efficacy for contraceptives, and efforts are in progress to examine and utilize this data. Such data, however, needs to be carefully considered as numerous sources of bias and/or confounders have been associated with RWD. For example, reporting and publication bias may be associated with the reporting of adverse events, and misclassification bias can perturb the usefulness of RWD [56,57]. Misclassification typically occurs due to the missingness of data. Missingness is a well-recognized limitation of RWD, and using datasets for pharmacokinetic studies outside their originally intended use case is often restricted by limited accuracy and/or documentation of factors such as dosing information, adherence, and treatment start or end dates [58,59]. Further “confounding by indication” may occur in RWD, with particular treatments or treatment regimens being associated with more severe disease states and, thus, worse outcomes [55]. Combined with the lack of randomization and blinding typically associated with RWD, its value in making causal inferences can be limited [56,57].

These limitations, however, can be managed through well-designed studies, propensity score matching, and probabilistic bias analyses; the use of multiple RWD sources further helps to build confidence in any results drawn [56,57]. In addition to this, findings may be further supported through validation with physiologically based pharmacokinetic (PBPK) models.

Validated PBPK models are available for LNG that explore various scenarios regarding dosing, co-administration of other drugs (perhaps with DDI potential), and specific patient populations [26,60,61]. Modifications to these models (e.g., dose route changed for implant scenario) and incorporation of covariate factors (e.g., specific patient characteristics, demographic factors, and co-medications/drug interactions) may be helpful to assess the impact of expected changes in exposure for a specific substance and any associated trends in safety or efficacy outcomes. For example, Lingenini et al. [22] have demonstrated that clinically significant lower exposures for a given oral LNG dose (100 or 150 μg) are predicted by PBPK modeling for subjects co-dosed with inducers of LNG metabolism (with a more marked reduction in high BMI subjects) and that these lower exposures would then be reflected in higher PI (i.e., lower efficacy) for these subjects according to the PI concentration-response relationship, with again a more marked effect with higher BMI. Recommendations can then be inferred for clinical practice (e.g., advising the use of backup contraceptive methods for particular patients according to body size, co-medications, etc.). Adaptation of such a PBPK model for application to LARC implant LNG used in conjunction with a LARC implant concentration-response relationship (such as that outlined in this paper) may allow similar predictions and recommendations for this alternate dosing route or for other patient populations. Once any adapted PBPK model framework applied in the LARC implant LNG scenario is constructed and validated with the use of specific observed data, the information in RWD datasets might then cross-validate the implied impacts of these changes in exposure on wider population safety and efficacy. Careful consideration needs to be given to the safety events being examined and how they are documented, particularly with respect to dosing information in RWD.

Another approach could include a more detailed exploration of available safety data; this may be extended to include unpublished or grey literature sources. However, care should be taken to ensure the quality of such data. Additional analysis of existing data may also be beneficial. This could include a thorough meta-analysis with forest plots or Kaplan–Meier plots illustrating time-to-event data for safety outcomes; however, given the sparsity of data, these are considered unlikely to be informative. A possible approach would be to pool the existing safety data to give an upper safety threshold. Regulatory input would be required to endorse this threshold, and collaboration should be included across the agencies.

## 4. Methods

### 4.1. Defining the LNG Exposure Bracket

#### 4.1.1. Defining the Efficacy Concentrations (Minimum Bracket)

The literature was reviewed to identify publications that described the efficacy of three LARC implant products as quantified by PI: Norplant, Sino Implant, and Jadelle. These three products were selected as levonorgestrel-releasing implants. Although the doses may differ between products, each product aims to provide low progestogen doses (40–50 μg/day) in the first year [62]. In parallel (if not available in the publication describing the efficacy), a further search was carried out to identify the plasma concentration exposure of LNG at times after implantation when the PI values were calculated and reported. The results are reported as timepoint-matched, parallel, observed pharmacokinetic and pharmacodynamic time courses for LNG following implantation of the different LARCs. These data were used to define concentration-response curves for the PI of the different LARC implants.

The profiles of PI effect vs. reported concentration were analyzed by fitting with Imax models via nonlinear regression in the R data analysis environment [63] to estimate Imax, IC_50,_ and IC_99_ values. The equations for the modeled relationship between Pearl Index and plasma LNG concentration (“conc”) and for calculation of IC_99_ were as follows (and with E_0_, the baseline PI in the absence of LNG concentration, fixed to a literature value—see results):

Pearl Index (PI) = E_0_ ∗ (1 − (I_max_ ∗ conc)/(IC_50_ + conc)) (1)

IC_99_ = (IC_50_ ∗ 0.99)/(I_max_ − 0.99) (2)

In the results section (Table 1), the IC99 values quoted in the table are calculated from the full precision of the parameter estimates from the curve fittings, which were quoted as rounded values in the table for reasons of style.

#### 4.1.2. Defining the Safety Concentrations (Maximum Bracket)

**Figure 3:**
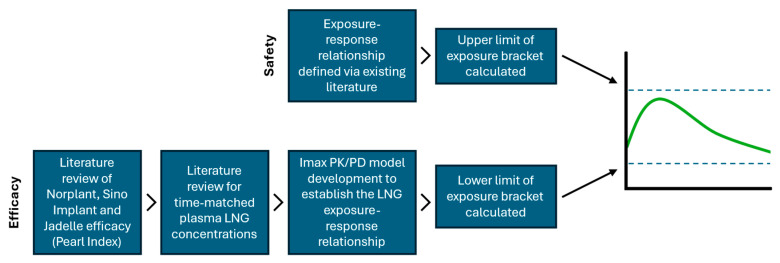
Proposed schema of work for defining the LNG exposure bracket.

Figure 3 shows the proposed schema of work for defining the LNG exposure bracket. Literature reviews were performed using the term “exposure-response of levonorgestrel”. More general searches were also performed using the term exposure-response for the safety of hormonal contraceptives, exposure-response for specific adverse events of interest (e.g., breakthrough bleeding, effects of bone mineral density), or reviews of the safety of hormonal contraceptives. Data was analyzed to consider whether a safe concentration could be defined for a number of the adverse events of interest, including weight gain, bleeding, and effects on bone mineral density.

### 4.2. Regulatory Precedence for the Exposure Matching Approach

The search for regulatory precedence is an important step in supporting both development strategies for new formulations and regulatory decision-making [64]. In order to consider current approaches for the development of long-acting products and determine the precedence for a bracketing approach, a search of EMA [65] and MHRA databases [66] was made. Instances were sought where a new long-acting product has been developed from an existing well-known product containing the same API but with a shorter duration of action. Information related to the entirety of the non-clinical and clinical pharmacology, efficacy, and safety data to support the application was extracted from the public assessment reports and analyzed. Examples were sought where extrapolation was used based on a known exposure-response relationship and similarity in PK exposure.

## 5. Conclusions

A bracketing approach based on maintaining plasma concentrations within predefined concentrations for efficacy and safety could be acceptable as per regulatory guidelines. This approach could avoid the need for expensive clinical and safety studies and, therefore, will significantly cut down on the time and cost of the development of new long-acting contraceptive products and other long-acting products getting to market. A review, however, of regulatory submissions in Europe and the UK shows limited precedence for such an approach. Notwithstanding, there are a number of examples demonstrating a limited non-clinical package and an abbreviated clinical package. When considering this approach for levonorgestrel, there appears to be a reasonable understanding of the minimal concentrations for efficacy, despite apparent differences between products, possibly due to poor/variable adherence. Maximum concentrations for safety are less well-defined. Adverse events are reasonably well understood, but the exposure-response understanding is currently not considered sufficient. Additional work is needed in this area, and could utilize RWD and systems pharmacology and/or PBPK modeling.

## Figures and Tables

**Table 1:** Summary of LNG Implant Literature Pharmacokinetic and Pharmacodynamic (PKPD) data, and parameter estimates from PKPD Imax Model Fittings.

Product/Source of Data	Years Since Implantation	Plasma PK Concentration (pg/mL)	Pearl Index (%)	Imax, % (%RSE)	IC50, pg/mL (%RSE)	IC99, pg/mL (calc.)
Norplant—PK: Croxatto (1993) [17], PD: Sivin (1988) [18]	5	290	1.1	101 (3.1)	5 (185)	301
	4	271	0.5			
	3	335	0.9			
	2	330	0.2			
	1	350	0.2			
Sino-Implant—PK and PD: Steiner (2019) [19]	5	-	4.59	117 (9.7)	45 (65)	245
	4	206	3.54			
	3	217	0.34			
	2	256	0.28			
	1	314	0			
Jadelle—PK: Sivin et al. (2001) [20], PD: FDA [21]	7	224	-	103 (0.1)	12 (0)	268
	6	258	-			
	5	279	0.88			
	4	271	-			
	3	280	0.13			
	2	312	0.1			
	1	340	0.08			

%RSE = percent relative standard error (precision) of the parameter estimate.

**Table 2:** Summary of non-clinical and clinical data from public assessment reports for long-acting products.

Product and Basis of Application	Non-Clinical Pharmacology	Clinical Pharmacology	Clinical Efficacy	Clinical Safety
**One Xeplion** [41] *Paliperidone palmitate* Article 8.3 From oral to monthly IM	PK studies in several species Full 6-month toxicology studies	Phase I clinical pharmacology study conducted in patients with schizophrenia. PK data collected from the Phase III studies for popPK analysis. IVIVC study performed. PK characteristics derived from studies with oral paliperidone also considered. PK report requested to compare data after treatment with paliperidone prolonged-release tablets and paliperidone palmitate LAI. PopPK modeling techniques were applied to support the comparison.	Four controlled, dose-finding studies Two flexible-dose, non-inferiority studies. Maintenance of effect was studied in one placebo-controlled, flexibly dosed, relapse prevention study and one flexible-dose, non-inferiority study. Following the CHMP assessment of the application for the indication of schizoaffective disorder approved in April 2011, the MAH committed to providing a long-term maintenance study on paliperidone palmitate	The safety database included population Phase II/III short-term trials, long-term trials, non-inferiority short and long-term trials, and clinical pharmacology trials in patients with schizophrenia.
**Trevicta** [42] *Paliperidone palmitate* Article 8.3 From 1-monthly to 3-monthly depots	Local tolerance in vivo study only	Single- and multiple-dose PK across the entire proposed dose range. PopPK model was developed based on data from the Phase I study and the Phase III study with sparse sampling.	Two Phase III studies	The clinical safety is based on results from 3 clinical studies in adults with schizophrenia.
**Okedi** [43] *Risperidone* Article 10.3 From oral to monthly IM	Pharmacology relies on information from the Risperdal tablets. Numerous PK studies conducted. Sub-chronic toxicity studies of 28 days (rabbit) and 31 days (dogs) Chronic (12-cycle, 30 days (rabbits, dogs)/cycle) toxicity studies	PK studied in one single-dose Phase I study. PopPK simulations support the doses.	Three Phase I studies. Four multiple-dose studies. Two comparative bioavailability studies in stable schizophrenia. One Phase II study in stable schizophrenia. One Phase III study in acute relapse of schizophrenia.	The adverse event profile of oral risperidone is well established.
**Zyphadera** [44] *Olazapine* Article 8.3 From daily tablets to fortnightly or monthly depots.	Primarily based on the findings reported in the dossier for oral olanzapine, as pharmacology is not expected to be altered by the route of administration. Absorption and exposure of olanzapine and/or palmoic acid studied following administration of depot in rats, rabbits, and dogs.	Only a pilot phase I study has been conducted on healthy volunteers. All PK pivotal studies have been conducted in patients. A total of 18 healthy volunteers and 412 patients were enrolled in five formal PK studies.	Phase III program is based on two main studies, one 8-week randomized placebo-controlled superiority study and one 24-week randomized active controlled versus oral olanzapine non inferiority study. A long-term, open-label study assessing safety, effectiveness, and PK was ongoing at the time of the opinion.	The total number of patients is 1778. At the time of database lock for this application, 445 patients had received at least 1 continuous year of treatment with Zyphadera.
**Abilify Maintena** [38] *Aripiprazole* Article 8.3 Oral to monthly IM for maintenance	Primarily consisted of studies previously conducted with oral or IM rapid solution for injection. The PK profile of IM aripiprazole depot and its metabolites was studied in rats. Repeat-dose toxicity studies up to 6 months in rats, 12 months in dogs, and 4 weeks in monkeys, and local tolerance studies in several species (rats, rabbits, dogs, and monkeys) were performed.	Phase I clinical pharmacology program has been conducted in patients with schizophrenia. PopPK analysis using data from Phase I and III studies (plus Phase I studies from the development of oral aripiprazole). Simulation of median aripiprazole concentration after administration of 400 mg IM aripiprazole depot, at dose initiation and steady-state, comparison to oral aripiprazole.	The clinical development included long-term studies investigating the maintenance of the effect. Maintenance was studied in one placebo-controlled withdrawal study and one active-controlled non-inferiority study (versus oral aripiprazole).	The safety database presented in the dossier included completed phase III controlled trials and all other trials. 1624 adult subjects with schizophrenia have been exposed to aripiprazole IM depot (15–400 mg).
**Rekambys** [45] *Rilpivirine (RPV)* Article 8.3 From daily oral to monthly IM	No new pharmacology data as the pharmacological profile of RPV LA is comparable to the approved oral RPV. Studies specifically performed with RPV LA include bridging toxicity and local tolerance studies, and studies performed with an excipient.	The clinical pharmacology characteristics of RPV LA were determined based on studies with the CAB + RPV regimen and studies with RPV LA alone. Studies with oral RPV that determined the clinical pharmacology of RPV included.	The clinical development program consists of 2 randomized, controlled pivotal Phase III studies, supported by 2 randomized, controlled Phase IIb studies. A Phase IIIb Study evaluating monthly and bimonthly dosing is ongoing.	Pooled Phase III studies. The safety Population consisted of 591 subjects randomized to CAB + RPV and 591 subjects randomized to CAR.
**Buvidal** [46] *Buprenorphine (BPN)* Article 10.3 From daily sublingual to weekly and monthly SC injection	The rat, dog, and minipig were chosen as species to evaluate the PK profile. Single and repeat dose studies performed. Toxicology program of single and repeat dose studies in the dog and 1 study in the minipig.	BPN PK following SC injection was investigated in 5 clinical studies. The PK following administration of weekly and monthly doses was compared with data obtained after administration of SL BPN and after a single IV injection. A popPK model of BPN was developed to evaluate the influence of covariates on the PK of BPN and for simulations of different treatment schedules.	The approved posology is supported by substantial clinical evidence, including PK, PD, efficacy, safety, and tolerability from studies including one Phase III study, as well as popPK modeling and simulation.	Safety is supported by clinical studies (*n* = 3214) using the reference product. The relative BA of BPN for CAM2038 provided plasma levels of BPN comparable to those of daily SL BPN. A total of 729 subjects were exposed to CAM2038 in the clinical program.
**Exparel** [47] *Bupivacaine* Article 8.3 Single-use injection with prolonged release	No new pharmacology studies. One study in guinea pigs to evaluate the effect of duration and site irritation. The applicant conducted PK studies. An abridged toxicological program was designed to support the MAA of Exparel.	Clinical PK data were obtained in 4 Phase II and 6 Phase III studies. Several healthy volunteer studies were conducted where Exparel was administered mainly via SC. Full PK profiling was performed in all PK studies. Two popPK analyses were conducted.	Efficacy from six Phase II, randomized, double-blind, multicenter, dose-finding studies and eleven Phase III, randomized, double-blind, multicenter studies. Efficacy assessed s as a field block for local analgesia (7/11) or as a peripheral nerve block(4/11).	Across 35 studies in the Exparel clinical development program, a total of 2321 individuals were exposed to Exparel (612 healthy subjects in Phase I studies, 18 special population subjects in a Phase I study, and 1645 subjects in the intended target population in Phase I, II and Phase III studies) Doses ranged from 9 to 665 mg.
**Bydureon** [48] *Exenatide* Section 505(b), FDA From twice-daily IM injection to weekly IM	Reproductive and developmental toxicity is available with exenatide given twice daily. A 104-week carcinogenicity study with exenatide extended-release in rats, dosed fortnightly by SC injection.		A 24-week, randomized, open-label trial was conducted to compare the safety and efficacy of BYDUREON to BYETTA (same active ingredient, different regime) in patients (*n* = 252).	Bydureon did not have adverse effects on blood pressure. These data were taken only from the 24-week trial described above.
**Lutrate** [49] *Leuprorelin acetate* Article 8.3 From monthly IM injection to tree-monthly IM injection	Assessment of secondary PD, safety pharmacology, PD drug interactions, or toxicology not conducted. A brief review of the toxicological profile, performed with a similar formulation, was provided. PK/PD studies were performed, which gave some information on local tolerance.	The PK profile was characterized in a subgroup of 30 patients with prostate cancer from 1 Phase III study. An IVIVC study was performed. A popPK model was developed. Clinically relevant covariates of interest were assessed.	One phase III clinical study (open-label, multicentre study, with no comparator group) with the new formulation. 163 male patients with prostate cancer were enrolled.	Of the 163 patients enrolled, 157 patients received two injections of leuprolide acetate 22.5 mg depot in a 3-month interval. Overall exposure for these patients was 44.17 mg of leuprolide.
**Zutectra** [50] *Hepatitis B immunoglobulin preparation* Article 8.3 From IV infusion to SC injection every 1 or 2 weeks	Limited non-clinical programs performed as standard non-clinical models were considered of limited relevance. The non-clinical program consisted of two local tolerance studies conducted with Zutectra as well as a study investigating single-dose toxicity and safety of Intratect.	A Phase I study to establish PK following SC administration compared to IM administration.	One phase III study (open one-arm study; *n* = 30) investigating the safety and efficacy of SC use in liver transplant. Data from supportive studies conducted with a different hepatitis B immunoglobulin preparation was also provided.	The safety of a product with SC administration is expected to be better than that of one of the same product for intravenous use. Lesser systemic exposure and lower propensity to induce a hypersensitivity response during the SC administration of Zutectra is anticipated to translate into a safety benefit.
**Sayana** [39] *DMPA* Article 8.3 From three-monthly IM injection to three-month SC injection	No new pharmacology or pharmacodynamic studies. A single SC dose in female rabbits was performed with thirteen weeks of follow-up.	Three phase I/II clinical pharmacology studies were completed: 1 dose-finding; 1 PK/PD in Asian women and 1 PK/PD including PD comparison for SC vs. IM)	The application is supported by two phase III efficacy and safety studies. Both multicentre, open-label, non-comparative, 1 year duration	3-year study to compare bone mineral density changes and evaluation of contraceptive efficacy (ongoing). Phase III comparison of SAYANA and leuprolide in endometriosis (ongoing)
**Sayanaject** [51] *DMPA* Three-monthly SC injection, change to the device	No new non-clinical studies are provided as the PD, PK, and toxicological properties are well known	One randomized open-label parallel group study of 68 healthy volunteers was used to assess the PK following different administration routes	No new clinical efficacy data were submitted	No new clinical safety data were submitted
**Mirena** [52,53,54] *Levonorgestrel* From 5-year IUD to 8-year IUD	No new data.	In vivo release rates were estimated using a popPK approach based on ex vivo residual content data. The popPK approach is using LNG residual content data from removed Mirena IUSs from 3 different clinical studies covering the complete 8-year period of use.	Contraceptive efficacy of Mirena during extended use (5–8 years) assessed in 362 women in the US in a Multi-centre, open-label, uncontrolled study	The study evaluated contraceptive efficacy, pharmacokinetics, and safety parameters.

Abbreviations: In vitro-in vivo correlation (IVIVC); Long-acting injectable (LAI); Pharmacokinetics (PK); Pharmacodynamics (PD); Population PK (popPK); Subcutaneous (SC); Intramuscular (IM); Medroxy-progesterone acetate (DMPA); Intrauterine device (IUD).

## Data Availability

Data is contained within the article.
